# Editorial: Reviews and recent advancements in animal welfare, volume II

**DOI:** 10.3389/fvets.2025.1685456

**Published:** 2025-10-01

**Authors:** Edward Narayan, Barbara Padalino

**Affiliations:** ^1^Faculty of Science and Engineering, Southern Cross University, Lismore, NSW, Australia; ^2^School of Education and Professional Studies, Griffith University, Gold Coast, QLD, Australia; ^3^Department of Agriculture and Food Sciences, University of Bologna, Bologna, Italy

**Keywords:** policy, welfare assessment, behavior, livestock, wildlife, pets

The second volume of the Research Topic entitled “Review in Animal welfare,” highlights the growing interest in protecting animal welfare, not only of livestock, but also of wild animals. This is important as animals both in the wild and in captive environments need protection and care. The RT includes 6 articles, three reviews and three research articles.

The three reviews address three different topics on farming animals, one on the housing systems of dairy calves, currently under possible legal revision in Europe, one on heat stress in beef cattle in Africa, highlighting the effects of global warming, and the last on Nile tilapia, the most farmed species of fish. Fish welfare is indeed one of the “hottest” topic in animal welfare science.

Plaugher and Cantor's scoping review (27 June 2025) examined the effects of pair housing on dairy calves, synthesizing findings from a wide range of studies. Their review highlighted consistent improvements in cognitive development, social interactions, and weight gain when calves were housed in pairs rather than individually. Importantly, they reported management strategies—such as feeding systems and pen design—that reduced cross-sucking, showing that welfare gains need not compromise farm productivity.

Slayi et al.'s systematic review (24 September 2024) focused on heat stress in sub-Saharan African feedlot cattle. Drawing on field trials, on-farm surveys, and climate modeling studies, they assessed interventions including shade provision, heat-tolerant breeds, and low-cost ventilation systems. Results pointed to reduced morbidity, better feed conversion, and improved animal behavior when climate-resilient infrastructure was used, underlining the intersection between animal welfare, food security, and community livelihoods.

Emam et al. (6 May 2025) turned attention underwater, reviewing the welfare of Nile tilapia, one of the world's most widely farmed fish. Through an analysis of experimental and observational studies on pain perception, stocking density, and slaughter methods, they concluded that tilapia—despite their hardiness—experience measurable stress and welfare challenges. The authors called for aquaculture to adopt the same science-based welfare benchmarks applied to terrestrial livestock.

Similarly, the three research articles tackle three topics of great interest, and it is not surprising, that two of them are on companion animals. Pet animals require basic welfare needs to ensure they are managed in a safe environment and humane manner. New scientific evidence has instead showed that companion animals are at the same risk of poor welfare as farmed animals (Sinclair et al. 2022). This topic warrants further investigation.

Schmid et al. (9 January 2025) investigated brachycephalic obstructive airway syndrome (BOAS) in French Bulldogs through anatomical measurements, respiratory function tests, and biomarker profiling. Their work produced an evidence-based severity grading system, providing breeders and veterinarians with a tool to improve breeding decisions and clinical management.

Hu et al. (2 December 2024) presented a technological breakthrough for companion animal welfare: a non-contact, video-based heart rate monitoring system. Tested in laboratory settings, the system accurately detected heart rates without restraining the animals, it can potentially be used to reduce stress during examinations and offers possibilities for telehealth applications. Being able to monitor HR during the vet check allows the vet to interpret signs of fear and discomfort and apply fear-free approaches, preventing animal-human injuries, often triggered by fear. So this new technology could be recommended to improve both human and animal health and welfare.

The final study by White et al. (2 October 2024) analyzed wildlife rehabilitation admissions in Finland, using multi-year datasets from rescue centers to identify leading causes such as vehicle collisions, disease outbreaks, and habitat loss. By linking patient outcomes with environmental and human activity patterns, the authors argued for preventive conservation measures, including urban planning that reduces wildlife-vehicle interactions and public education to address emerging threats.

As veterinary science broadens its scope—from calves on dairy farms to tilapia in aquaculture, from companion animals at home to wildlife in rescue centers—three interwoven themes emerge: improving animal welfare, embracing technological innovation, and enhancing resilience against environmental stressors. The following papers exemplify this multidisciplinary synergy, each contributing critical evidence and perspective.

The collective insights of these papers paint a holistic vision for veterinary science—one that champions welfare across species, leverages emerging technologies, and fortifies resilience in the face of environmental change. By integrating husbandry innovations, breeding reforms, remote health monitoring, and conservation-oriented practices, we edge closer to sustainable, ethical, and evidence-based stewardship of animal lives.

Collectively, these studies span farmed, companion, and wild animals, yet all support the same underlying voice: welfare improvement requires scientific evidence to guide practical interventions. Whether through better housing, climate adaptation, technology, breeding reform, or habitat protection, the research points to potential integrated solutions that advance both animal wellbeing and broader societal goals. All these research show promise and require further research in practical settings.

The articles in this Research Topic expand our understanding of animal welfare science and provide evidence that can inform the revision of current welfare policies. They underline the importance of training all those involved in animal-related activities from farming to wildlife rehabilitation in best practices, including stress-free handling and evidence-based approaches grounded in animal learning theory. Monitoring behavioral and physiological parameters, alongside the adoption of innovative technologies, can help safeguard welfare on farms, in the wild, and during veterinary care. Like all rigorous research, these studies raise new questions for future investigation. We remain committed to advancing evidence-based guidelines in animal welfare through the *Frontiers* open-access platform and encourage future article collections to explore animal health and welfare in a holistic, interdisciplinary manner.

